# Association between the C-reactive protein–triglyceride glucose index and postoperative acute kidney injury after elective abdominal surgery: a retrospective cohort study

**DOI:** 10.3389/fmed.2026.1933511

**Published:** 2026-10-26

**Authors:** Yu qi Zhong, Binbin Wang, Xiuxia Gu, Rui Zhang, Fangli Yue, Yajuan Zhao

**Affiliations:** Department of Anesthesiology, Weifang People’s Hospital, Weifang, China

**Keywords:** C-reactive protein, elective abdominal surgery, inflammation, postoperative acute kidney injury, triglyceride glucose index

## Abstract

**Background:**

Postoperative acute kidney injury (PO-AKI) is a common and serious complication following abdominal surgery and is strongly associated with poor prognosis. Traditional diagnostic markers lack sensitivity. The novel C-reactive protein–triglyceride glucose index (CTI) has emerged as an innovative composite marker for evaluating metabolic-inflammatory dysregulation, comprehensively integrating markers of insulin resistance and systemic inflammation. However, its relationship with PO-AKI risk remains unclear.

**Methods:**

This single-center retrospective study included adult patients who underwent elective major abdominal surgery between April 2020 and April 2025. The preoperative CTI was the primary exposure variable. The primary outcome was the incidence of AKI within 7 days post-surgery, defined according to the KDIGO creatinine criteria. Multivariable logistic regression was used to assess the association between CTI and PO-AKI, and restricted cubic splines (RCS) were applied to examine dose–response relationships. Receiver operating characteristic (ROC) curve analysis was performed to compare the predictive performance of CTI and the TyG index for PO-AKI. Subgroup analyses and sensitivity analyses were conducted to evaluate the robustness of the findings.

**Results:**

A total of 4,658 patients were included, of whom 222 (4.77%) developed PO-AKI. After full adjustment for confounders, higher CTI was significantly associated with increased PO-AKI risk (OR = 1.28, 95% CI: 1.09–1.52). Compared with patients in the lowest CTI quartile, those in the highest quartile had a significantly higher risk (OR = 1.86, 95% CI: 1.12–3.11). RCS analysis revealed a linear positive association between CTI and PO-AKI. The area under the ROC curve (AUC) for CTI was significantly greater than that for the TyG index alone (0.643 vs. 0.587; *P* = 0.0003). Moreover, CTI values above approximately 9.5 were associated with prolonged postoperative hospital stay.

**Conclusions:**

The preoperative CTI, a surrogate marker for the systemic immuno-metabolic phenotype, is an independent risk factor for PO-AKI after abdominal surgery. Notably, a CTI threshold of 9.5 serves as a cclinical decision node, offering superior predictive value for prolonged care trajectories and informing targeted perioperative prehabilitation strategies.

## Introduction

The incidence of acute kidney injury (AKI) has risen steadily in recent years and remains substantially high. AKI is now the fourth most common postoperative complication, following bleeding, surgical site infection, and arrhythmia (1). It can impair the function of multiple vital organs—including the gastrointestinal tract, liver, lungs, and heart—and significantly affects patient prognosis (2). AKI is consistently associated with elevated mortality and morbidity, prolonged and more complex hospitalizations, and increased healthcare costs. Even modest increases in serum creatinine have been linked to higher short- and long-term mortality (3, 4).

The etiology of postoperative AKI (PO-AKI) is multifactorial. Hypoperfusion, inflammatory injury, and neuroendocrine responses to surgical stress are key mechanisms that compromise renal perfusion (5). In patients undergoing abdominal surgery, factors such as substantial fluid shifts, impaired renal blood flow, systemic inflammation, and elevated intra-abdominal pressure may contribute to the development of PO-AKI (6). Previous retrospective studies have reported PO-AKI incidence rates ranging from 1.8% to 39.3% following abdominal procedures (7). Early identification and prediction of AKI are therefore of considerable clinical importance. However, conventional markers—serum creatinine and urine output—lack sensitivity, often leading to delayed diagnosis and intervention (1, 8).

Emerging biomarkers offer new opportunities for early AKI detection (9). For instance, urinary biomarkers such as NGAL, KIM-1, and [TIMP-2] × [IGFBP7] enable the detection of subclinical renal tubular injury before functional impairment becomes evident by serum creatinine criteria (10, 11). However, these biomarkers are predominantly measured in the postoperative period and primarily reflect downstream structural tubular injury. In contrast, the CTI, a novel composite metric incorporating CRP and the TyG index, captures both systemic metabolic status and the synergistic interaction between inflammation and metabolism (12). Rather than merely functioning as a mathematical combination, CTI effectively quantifies the preoperative “metabolic-inflammatory dysregulation”. By capturing the upstream systemic immuno-metabolic vulnerability, CTI allows for risk stratification before surgery, thereby complementing the early postoperative diagnostic value of urinary biomarkers. The study aimed to evaluate the ability of the preoperative CTI to predict PO-AKI in patients undergoing elective abdominal surgery, potentially offering new insights into risk stratification and perioperative management.

## Methods

### Study design and population

This retrospective observational study was approved by the Institutional Review Committee of Weifang People's Hospital, which waived the requirement for informed consent.

Data were extracted from the electronic medical record system of a tertiary hospital in China, including demographic information, anesthesia records, laboratory results, and diagnostic data. The hospital has 3,600 beds and performed 124,800 surgeries in 2024. We included adult patients (aged ≥18 years) who underwent elective major abdominal surgery under general anesthesia between April 2020 and April 2025. Abdominal surgery was defined as any intra-abdominal procedure involving the stomach, intestines, liver, pancreas, or spleen (13).

Exclusion criteria were: (1) American Society of Anesthesiologists (ASA) class V or VI; (2) age < 18 years; (3) absence of pre- or postoperative serum creatinine measurements; (4) missing preoperative fasting glucose, triglyceride, or CRP data; (5) AKI diagnosed within 30 days before surgery, or end-stage renal disease (defined as a history of renal replacement therapy or kidney transplantation, or an estimated glomerular filtration rate < 15 mL/min/1.73 m^2^ based on the modified Chinese diet–renal disease equation) (14).

### Assessment of CTI

The CTI was calculated using preoperative blood samples as previously described (9). CRP, triglycerides, and fasting glucose were measured, with triglycerides and glucose used to estimate insulin resistance. All preoperative variables were based on the most recent measurements within 30 days before surgery (15). CTI was defined as: 0.412 × ln(CRP) (mg/dL) + ln([triglyceride (mg/dL) × fasting glucose (mg/dL)]/2) (12).

### Covariates

Based on demographic characteristics, lifestyle factors, clinical status, and prior literature, we selected potential confounders associated with PO-AKI. Variables with >30% missing data were excluded. The final set included sex, age, body mass index (BMI), smoking and alcohol use, hypertension, hyperlipidemia, diabetes, ASA classification, platelet count, eGFR, prothrombin time, aspartate aminotransferase, alanine aminotransferase, total bilirubin, globulin, high-density lipoprotein, serum potassium, calcium, sodium, surgical duration, and intraoperative total fluid output. BMI was calculated as weight (kg) divided by height squared (m^2^) (16). Hypertension, diabetes, and hyperlipidemia were defined based on medical history or current use of relevant medications (17).

### Outcomes

The primary outcome was AKI occurring within 7 days after surgery, defined according to the Kidney Disease: Improving Global Outcomes (KDIGO) creatinine criteria (18): an increase in serum creatinine ≥ 0.3 mg/dL (26.5 μmol/L) within 48 h, or an increase to ≥ 1.5 times the baseline value within the prior 7 days. The most recent preoperative creatinine value was used as baseline. Postoperative hospital stay was a secondary outcome.

### Statistical analysis

All analyses were performed using R software (version 4.4.3). Two-tailed *P* values < 0.05 were considered statistically significant. Missing covariate data were imputed using random forest imputation.

Normally distributed continuous variables were presented as mean ± standard deviation, non-normally distributed variables as median with interquartile range (IQR). Categorical variables were expressed as frequencies and percentages. Group comparisons were performed using unpaired t-tests, Mann–Whitney U tests, *χ*^2^ tests, or Fisher's exact tests, as appropriate.

Logistic regression models were used to evaluate the association between CTI and PO-AKI, with results reported as odds ratios (OR) and 95% confidence intervals (CI). Three models were constructed: Model 1 (unadjusted); Model 2 (adjusted for age, sex, smoking, and alcohol use); and Model 3 (further adjusted for BMI, diabetes, hypertension, platelet count, eGFR, prothrombin time, aspartate aminotransferase, alanine aminotransferase, total bilirubin, ASA class, globulin, high-density lipoprotein, serum potassium, calcium, sodium, surgical duration, and intraoperative total fluid output). CTI was analyzed both as a continuous variable (per 1-unit increase) and categorized by quartiles (with the lowest quartile as reference). Trend tests were performed using the median value of each quartile. Collinearity was assessed using tolerance and variance inflation factor (VIF), with VIF < 5 indicating no significant collinearity.

Restricted cubic spline (RCS) regression with three knots at the 10th, 50th, and 90th percentiles was used to explore potential nonlinear relationships between CTI and PO-AKI. Receiver operating characteristic (ROC) curves were generated to compare the predictive performance of CTI and the TyG index, with areas under the curve (AUC) calculated and compared using the DeLong test. Subgroup analyses were conducted stratified by sex, age (<60 vs. ≥60 years), BMI (<24 vs. ≥24 kg/m^2^), smoking (yes/no), alcohol use (yes/no), hypertension (yes/no), and diabetes (yes/no), with interaction tests performed to assess effect modification.

To further evaluate the robustness of the findings, a sensitivity analysis was performed excluding patients with pre-existing renal impairment, defined as an eGFR < 60 mL/min/1.73 m^2^ according to KDIGO guidelines. The multivariable logistic regression model was repeated in this restricted cohort.

## Results

### Study population

A total of 8,175 patients who underwent abdominal surgery met the initial inclusion criteria. After applying exclusion criteria, 4,658 patients were included in the final analysis (Figure 1). Of these, 222 (4.77%) developed PO-AKI within 7 days post-surgery.

**Figure 1:**
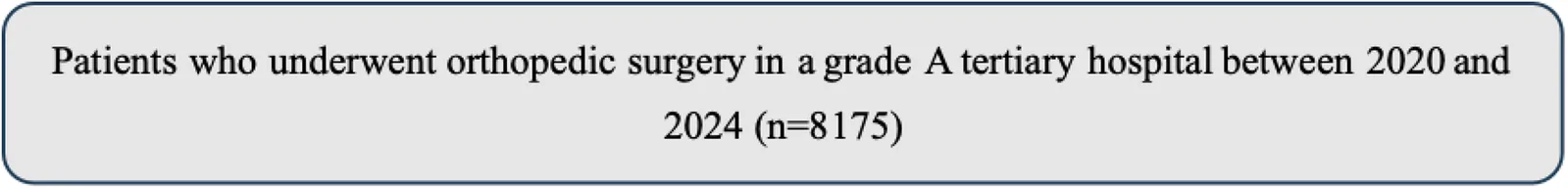
Flowchart of patient selection.

### Baseline characteristics

Patients who developed PO-AKI were older, more likely to smoke, and had higher prevalence of hypertension and diabetes (all *P* < 0.05). They also exhibited lower hemoglobin, white blood cell count, albumin, alanine aminotransferase, total cholesterol, low-density lipoprotein, and high-density lipoprotein levels, but higher levels of uric acid, serum creatinine, CRP, and serum potassium. Prothrombin time and postoperative hospital stay were longer in the PO-AKI group. ASA classification also differed significantly between groups. Both the TyG index and CTI were significantly elevated in patients who developed PO-AKI (Table 1).

**Table 1:** Baseline characteristics of participants by grounps of AKI.

Characteristic	Overall	AKI (NO)	AKI (YES)	*P* value
n (%)	4,658	4,436 (95.2)	222 (4.8)	
Female, *n* (%)	2,267 (48.7)	2,153 (48.5)	114 (51.4)	0.413
Age, years	58 (45, 68)	58 (45, 67)	67 (57, 73)	<0.001
BMI, kg/m2	24.2 (21.8, 26.6)	24.1 (21.7, 26.6)	24.6 (22.2, 27.3)	0.055
Smoking, *n* (%)	1,121 (24.1)	1,055 (23.8)	66 (29.7)	0.043
Alcohol consumption, *n* (%)	1,072 (23.0)	1,019 (23.0)	53 (23.9)	0.755
Diabetes, *n* (%)	544 (11.7)	487 (11.0)	57 (25.7)	<0.001
Hypertension, *n* (%)	1,325 (28.4)	1,201 (27.1)	124 (55.9)	<0.001
Hyperlipemia, *n* (%)	49 (1.1)	44 (1.0)	5 (2.3)	0.072
Hemoglobin, g/L	127 (115, 140)	127 (116, 140)	116 (101, 129)	<0.001
Platelet, ×10^9^	242 (202, 294)	242 (202, 292)	255 (192, 318)	0.276
White blood cell, ×10^9^	7.2 (5.9, 9.1)	7.2 (5.9, 9.0)	7.8 (6.1, 11.2)	<0.001
Total protein, g/l	66.3 (62.7, 70.1)	66.3 (62.7, 70.0)	66.9 (62.6, 72.0)	0.078
Albumin, g/L	38.7 (36.1, 41.1)	38.8 (36.2, 41.2)	36.2 (32.2, 39.0)	<0.001
Triglyceride, mg/dL	113 (82, 162)	113 (81, 161)	121 (88, 173)	0.111
Glucose, mg/dL	92.5 (83.5, 109.1)	92.1 (83.5, 108.2)	102.2 (85.6, 147.8)	<0.001
AST, U/L	17 (14, 22)	17 (14, 22)	17 (14, 23)	0.545
ALT, U/L	16 (11, 25)	16 (12, 25)	13 (9, 23)	<0.001
Creatinine, μmol/l	67.0 (56.0, 81.0)	67.0 (56.0, 80.0)	81.0 (60.8, 157.5)	<0.001
Total bilirubin, µmol/L	9.6 (7.0, 13.6)	9.6 (7.0, 13.6)	9.6 (6.6, 12.7)	0.312
TC, mg/dL	177 (150, 206)	177 (152, 206)	163 (130, 203)	<0.001
LDL, mg/dL	113 (92, 134)	113 (93, 134)	80 (46, 122)	<0.001
HDL, mg/dL	106 (52, 142)	108 (53, 143)	106 (52, 142)	<0.001
UA, µmol/L	342 (277, 416)	341 (276, 414)	363 (295, 464)	<0.001
CRP, mg/L	3.9 (1.2, 20.3)	3.8 (1.1, 18.9)	9.5 (2.5, 53.0)	<0.001
Prothrombin time, s	10.8 (10.2, 11.4)	10.8 (10.2, 11.4)	11.1 (10.3, 12.1)	<0.001
K^+^, mmol/L	4.0 (3.8, 4.2)	4.0 (3.8, 4.2)	4.1 (3.8, 4.6)	<0.001
Na^+^, mmol/L	141 (139, 142)	141 (139, 142)	141 (139, 142)	0.284
Ca ^+^ ^2^, mmol/L	2.3 (2.2, 2.3)	2.3 (2.2, 2.3)	2.2 (2.2, 2.3)	0.122
ASA *n* (%)				<0.001
I	655 (14.1)	648 (14.6)	7 (3.2)	
II	3,188 (68.4)	3,068 (69.2)	120 (54.1)	
III	774 (16.6)	690 (15.6)	84 (37.8)	
IV	41 (0.9)	30 (0.7)	11 (0.2)	
Intraoperative total fluid out, mL	1,000 (600. 1,350)	1,000 (600. 1,350)	975 (500. 1,300)	0.170
Stay after surgery, days	10 (8, 14)	10 (8, 14)	12 (8, 19)	<0.001
Procedure duration, min	129.0 (96.0, 174.3)	130.0 (96.0, 164,3)	120.0 (86.5, 164,3)	0.050
TyG	8.6 (8.2, 9.1)	8.6 (8.2, 9.1)	8.8 (8.4, 9.4)	<0.001
CTI	9.3 (8.6, 10.0)	9.3 (8.6, 10.0)	9.8 (9.1, 10.6)	<0.001

AKI, acute kidney injury; BMI, body mass index; WBC, white blood cell; AST, aspartate aminotransferase; ALT, alanine aminotransferase; TBIL, total bilirubin; TC, total cholesterol; LDL, low-density lipoprotein; HDL, high-density lipoprotein; K^+^, serum potassium; Ca^+^, calcium; Na^+^, sodium; ASA, American Society of Anesthesiologists Physical Status Classification System, UA, uric acid; CRP, C-reactive protein; CTI, C-reactive protein-triglyceride glucose index.

### Association between CTI and PO-AKI

In all logistic regression models, CTI was positively associated with PO-AKI. Per 1-unit increase in CTI was associated with an OR of 1.24 (95% CI: 1.10–1.41) in Model 1, 1.26 (95% CI: 1.11–1.45) in Model 2, and 1.28 (95% CI: 1.09–1.52) in the fully adjusted Model 3. When CTI was analyzed by quartiles, the risk of PO-AKI increased progressively with higher quartiles. Compared with the lowest quartile (Q1), the ORs for PO-AKI were 1.31 (95% CI: 0.78–2.18) for Q2, 1.69 (95% CI: 1.03–2.76) for Q3, and 1.86 (95% CI: 1.12–3.11) for Q4 (*P* for trend = 0.004) (Table 2).

**Table 2:** Logistic regression analysis for the association between different classes of CTI and AKI.

CTI Category	Model1		Model2		Model3	
	OR (95%CI)	*P* value	OR (95%CI)	*P* value	OR (95%CI)	*P* value
Q1	Reference		Reference		Reference	
Q2	1.60 (0.97–2.63)	0.066	1.40 (0.85–2.32）	0.186	1.31 (0.78–2.18)	0.309
Q3	2.42 (1.52–3.86)	<0.001	1.90 (1.19–3.05）	0.008	1.69 (1.03–2,76)	0.038
Q4	3.84 (2.47–5.98)	<0.001	2.87 (1.83–4.50）	<0.001	1.86 (1.12–3.11)	0.018
*P* for trend		<0.001		<0.001		0.004
Per SD	1.24 (1.09–1.41）	0.001	1.26 (1.11–1.45）	<0.001	1.28 (1.09–1.52）	0.004

Model 1: unadjusted. Model 2: adjusted for age, gender, smoking, alcohol. Model 3: model 2 + further adjusted for body mass index, diabetes, hypertention, platelet, prothrombin time, aspartate aminotransferase, alanine aminotransferase, total bilirubin, American Society of Anesthesiologists Physical Status Classification System, Globulin, high-density lipoprotein, serum potassium, calcium, sodium, procedure duration, intraoperative total fluid out, eGFR.

RCS analysis confirmed a linear dose–response relationship between CTI and PO-AKI (*P* for nonlinearity = 0.671) (Figure 2). ROC analysis showed that CTI had superior predictive ability for PO-AKI (AUC = 0.643, 95% CI: 0.630–0.657) compared to the TyG index alone (AUC = 0.587, 95% CI: 0.572–0.601; *P* for difference = 0.0003) (Figure 3).

**Figure 2:**
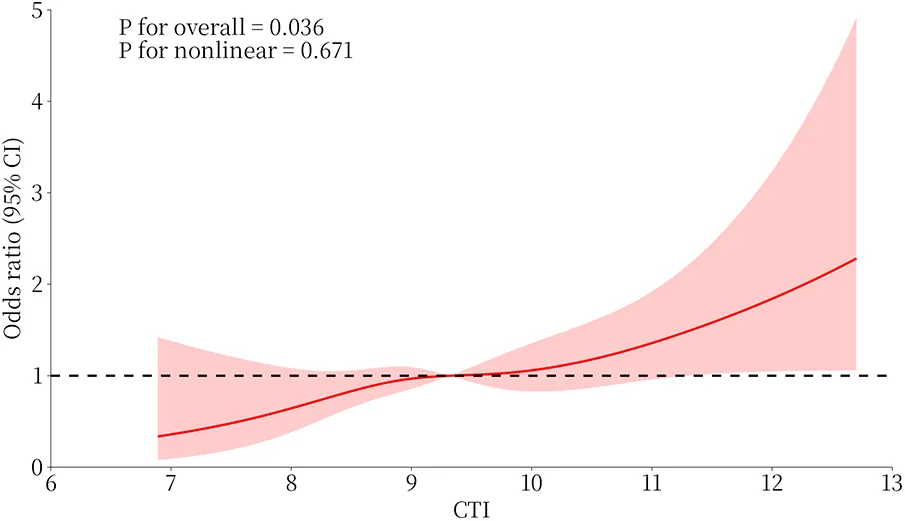
Restricted cubic spline analysis of the association between CTI and PO-AKI. Adjusted for all covariates in Model 3. Solid line represents adjusted odds ratios; dashed lines represent 95% confidence intervals.

**Figure 3:**
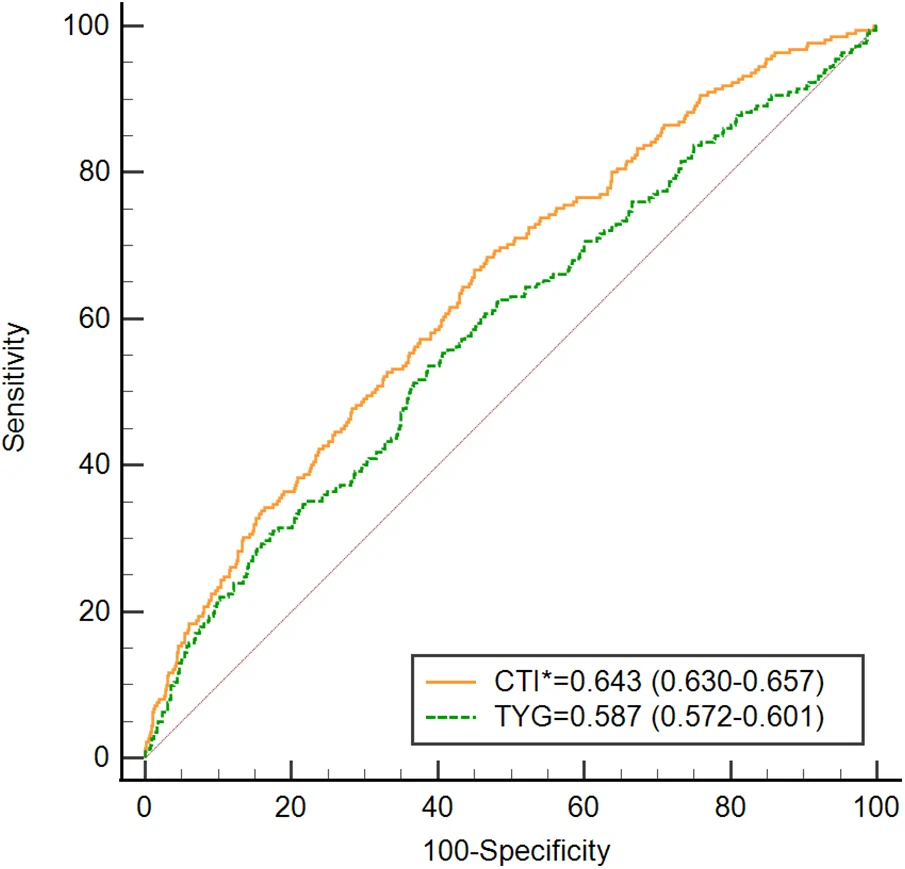
Receiver operating characteristic curves for CTI and tyG index in predicting PO-AKI.

### Subgroup analyses

The positive association between CTI and PO-AKI remained consistent across all predefined subgroups, including sex, age, BMI, smoking, alcohol use, hypertension, and diabetes. No significant interactions were observed (all *P* for interaction > 0.05) (Supplementary Table S1).

### Sensitivity analysis

To further assess the robustness of our findings, we performed a sensitivity analysis excluding patients with pre-existing renal impairment (eGFR < 60 mL/min/1.73 m^2^). In this restricted cohort, the positive association between CTI and PO-AKI remained statistically significant. Per 1-unit increase in CTI, the adjusted OR was 1.28 (95% CI: 1.05–1.56, *P* = 0.013) in the fully adjusted model. When analyzed by quartiles, the ORs for PO-AKI across increasing quartiles were 1.35 (95% CI: 0.77–2.39), 1.79 (95% CI: 1.03–3.10, and 2.19 (95% CI: 1.23–3.89), with a significant trend (*P* for trend = 0.013). These results are presented in Supplementary Table S2.

### CTI and postoperative hospital stay

RCS analysis revealed a nonlinear association between CTI and postoperative hospital stay (*P* for nonlinearity = 0.013). Below a CTI threshold of approximately 9.5, no significant association with hospital stay was observed. Above this threshold, higher CTI was significantly associated with prolonged hospitalization (Figure 4).

**Figure 4:**
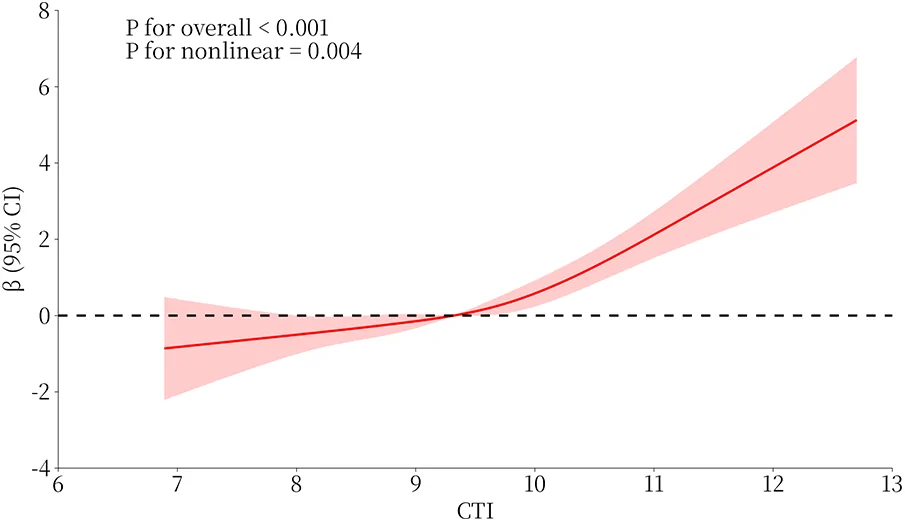
Restricted cubic spline analysis of the association between CTI and postoperative hospital stay. Adjusted for all covariates in Model 3. Solid line represents adjusted β coefficients; dashed lines represent 95% confidence intervals.

## Discussion

This large retrospective cohort study is the first to demonstrate a significant and independent association between the preoperative CTI and the risk of PO-AKI after elective abdominal surgery. The positive linear relationship persisted after comprehensive adjustment for confounders and remained robust across multiple subgroups. Furthermore, CTI exhibited superior predictive performance compared to the TyG index alone, suggesting that the integration of inflammatory and metabolic markers enhances risk stratification.

The CTI, originally developed by Ruan et al. for prognostic assessment in cancer patients, combines CRP—a marker of systemic inflammation—with the TyG index, a surrogate for insulin resistance (19). Both components have been individually linked to AKI risk. Elevated TyG index has been associated with increased AKI incidence in critically ill patients with heart failure and in ICU populations, with linear dose–response relationships reported (20, 21). Similarly, higher CRP levels have been associated with accelerated kidney function decline and increased AKI risk (22). Given the well-established interplay between insulin resistance and inflammation in the pathogenesis of vascular and renal injury, a composite index capturing both domains may offer a more comprehensive risk assessment than either marker alone. In addition, urinary biomarkers such as NGAL, KIM-1, and [TIMP-2] × [IGFBP7] have been proposed for detecting subclinical renal tubular injury before functional impairment becomes apparent; however, they are typically measured postoperatively and primarily reflect downstream structural injury. These biomarkers are therefore complementary to preoperative CTI, which captures upstream immuno-metabolic vulnerability (10, 11).

The enhanced clinical utility of the CTI index compared to isolated TyG or CRP biomarkers arises from its synergistic integration. Rather than merely functioning as a mathematical combination, CTI effectively quantifies the preoperative “metabolic-inflammatory dysregulation” (23). While isolated use of TyG or CRP captures singular pathological dimensions (insulin resistance or systemic inflammation, respectively), CTI's dual-marker framework reveals cross-system interactions, comprehensively capturing the synergistic immuno-metabolic phenotype that may drive endothelial dysfunction and renal hypoperfusion.

The uric acid/creatinine ratio (UCR) has been recognized as a biomarker of adverse outcomes in various clinical settings (24, 25). UCR and CTI reflect distinct pathophysiological dimensions: UCR primarily reflects renal tubular handling of uric acid and purine metabolism, whereas CTI captures systemic immuno-metabolic dysregulation. Therefore, these two indices may provide complementary information, and future studies are warranted to evaluate whether combining UCR with CTI could further improve risk stratification for PO-AKI.

The precise mechanisms linking an elevated CTI to PO-AKI may involve the synergistic amplification of surgical stress by pre-existing immune-metabolic dysregulation. Inflammatory activation, such as that assessed by the neutrophil-to-lymphocyte ratio, has been independently associated with subclinical atherosclerosis in high-risk subjects (26), and changes in lipid burden have been associated with modifications in the mechanical vascular phenotype assessed by pulse wave velocity (27). These observations support a broader interaction between metabolic, inflammatory, and vascular pathways, but also highlight the importance of distinguishing circulating risk markers from direct measures of vascular or organ-specific injury. Insulin resistance and chronic systemic inflammation may compromise microvascular endothelial integrity and may prime renal tubular epithelial cells for profound oxidative stress (28, 29). The convergence of impaired glucose utilization and lipid dysregulation—reflected by the triglyceride component of the CTI—may exacerbate the accumulation of reactive oxygen species (ROS) and may drive aggressive lipid peroxidation in the renal microenvironment (30, 31). During the ischemia-reperfusion phase typical of major abdominal surgery, this hostile metabolic-inflammatory milieu may overwhelm the endogenous antioxidant defense systems (6, 32, 33). This depletion of antioxidant capacity may contribute to regulated cell death cascades, including ferroptosis, potentially culminating in irreversible acute tubular necrosis (34). Thus, CTI may act as an upstream sensor for this molecular vulnerability.

Beyond its diagnostic accuracy, our study uncovered a potentially actionable clinical threshold. The restricted cubic spline analysis revealed a significant nonlinear relationship between CTI and postoperative hospital stay, with a distinct inflection point at approximately 9.5. This identifies CTI not just as a risk factor, but as a potential “clinical decision node.” Patients presenting with a CTI above 9.5 may exhibit a severe immuno-metabolic phenotype that may predispose them to prolonged recovery and amplified renal injury. Consequently, calculating the CTI during standard preoperative assessment may allow clinicians to triage high-risk patients for targeted prehabilitation—such as strict glycemic control, lipid-lowering therapies, and prophylactic anti-inflammatory strategies—prior to elective abdominal surgery (35).

### Limitations

Several limitations should be acknowledged. First, this was a single-center study, which may limit generalizability. Second, despite extensive adjustment for confounders, including preoperative eGFR, residual confounding cannot be excluded due to the observational design. Third, exclusion of patients with missing creatinine data may have introduced selection bias. Fourth, longitudinal changes in insulin resistance and inflammation were not assessed, precluding analysis of CTI trajectories. Fifth, urinary biomarkers of subclinical renal tubular injury were not measured in this cohort; therefore, we could not directly compare the predictive value of CTI with these biomarkers, and our findings should be interpreted in this context. Finally, the observational nature precludes causal inference.

## Conclusions

The preoperative CTI, a composite index integrating insulin resistance and systemic inflammation, is independently associated with PO-AKI after elective abdominal surgery and showed a higher AUC than the TyG index alone. These findings suggest that CTI may serve as a complementary biomarker for risk stratification and may inform perioperative strategies aimed at reducing AKI risk. Further prospective studies are warranted to validate these results and elucidate underlying mechanisms.

## Data Availability

The original contributions presented in the study are included in the article/Supplementary Material, further inquiries can be directed to the corresponding author.
