# Supplementary material for: Association between the C-reactive protein–triglyceride glucose index and postoperative acute kidney injury after elective abdominal surgery: a retrospective cohort study
Source: Front Med (Lausanne). 2026 Oct 26;13:1933511. doi: 10.3389/fmed.2026.1933511 (PMC13745549; doi:10.3389/fmed.2026.1933511)
Supplement: Supplementary file 1 [file Table1.doc]

Supplementary Table 1 Association of CTI with the risk of AKI stratified by different factors

| Subgroups | n (%) | OR (95%CI) | *P* valve | *P* for interaction |
| --- | --- | --- | --- | --- |
|  |  |  |  |  |
| All patients | 4658 (100.00) | 1.38 (1.19 ~ 1.59) | <0.001 |  |
| Gender |  |  |  | 0.100 |
| Male | 2267 (48.67) | 1.47 (1.20 ~ 1.78) | <0.001 |  |
| Female | 2391 (51.33) | 1.27 (1.02 ~ 1.59) | 0.033 |  |
| Age |  |  |  | 0.054 |
| <60 | 2496 (53.59) | 1.46 (1.14 ~ 1.87) | 0.003 |  |
| ≥60 | 2162 (46.41) | 1.34 (1.12 ~ 1.60) | 0.001 |  |
| BMI |  |  |  | 0.324 |
| ≤23.9 | 2212 (47.49) | 1.17 (0.95 ~ 1.44) | 0.133 |  |
| >23.9 | 2446 (52.51) | 1.65 (1.34 ~ 2.02) | <0.001 |  |
| Diabetes |  |  |  | 0.118 |
| No | 4114 (88.32) | 1.50 (1.27 ~ 1.77) | <0.001 |  |
| Yes | 544 (11.68) | 1.15 (0.86 ~ 1.54) | 0.336 |  |
| Hypertensive |  |  |  | 0.626 |
| No | 3333 (71.55) | 1.27 (1.03 ~ 1.56) | 0.024 |  |
| Yes | 1325 (28.45) | 1.48 (1.20 ~ 1.83) | <0.001 |  |
| Smoking |  |  |  | 0.987 |
| No | 3537 (75.93) | 1.41 (1.18 ~ 1.69) | <0.001 |  |
| Yes | 1121 (24.07) | 1.35 (1.05 ~ 1.73) | 0.018 |  |
| Alcohol consumption |  |  |  | 0.539 |
| No | 3586 (76.99) | 1.38 (1.17 ~ 1.64) | <0.001 |  |
| Yes | 1072 (23.01) | 1.43 (1.07 ~ 1.91) | 0.016 |  |
| OR: Odds Ratio, CI: Confidence Interval | | | | |

**Supplementary Table 2 Sensitivity analysis excluding patients with pre-existing renal impairment: Association between CTI and PO-AKI.**

|  | **Model1** |  |  | **Model2** |  |  | **Model3** |  |  |
| --- | --- | --- | --- | --- | --- | --- | --- | --- | --- |
|  | **OR(95%CI)** | **P value** |  | **OR(95%CI)** | **P value** |  | **OR(95%CI)** | **P value** |  |
| Q1 | Reference |  |  | Reference |  |  | Reference |  |  |
| Q2 | 1.54 (0.88-2.69) | 0.128 |  | 1.36 (0.78-2.38） | 0.282 |  | 1.35 (0.77-2.39) | 0.297 |  |
| Q3 | 2.09 (1.23-3.55) | 0.006 |  | 1.64 (0.96-2.80） | 0.069 |  | 1.79 (1.03-3.10) | 0.040 |  |
| Q4 | 2.76 (1.66-4.59) | <0.001 |  | 2.02 (1.20-3.38） | 0.008 |  | 2.19 (1.23-3.89) | 0.008 |  |
| P for trend |  | <0.001 |  |  | 0.012 |  |  | 0.013 |  |
| Per SD | 1.38 (1.18-1.62） | <0.001 |  | 1.24 (1.05-1.46） | 0.012 |  | 1.28 (1.05-1.56） | 0.013 |  |

Pre-existing renal impairment was defined as preoperative serum creatinine > 137 μmol/L in males and > 104 μmol/L in females (corresponding to an eGFR < 60 mL/min/1.73 m² according to KDIGO guidelines). Model 1: unadjusted. Model 2: adjusted for age, gender, smoking, alcohol.Model 3: model 2+further adjusted for body mass index, diabetes, hypertention, platelet, prothrombin time, aspartate aminotransferase, alanine aminotransferase, total bilirubin, American Society of Anesthesiologists Physical Status Classification System, Globulin, high-density lipoprotein, serum potassium, calcium, sodium, procedure duration, intraoperative total fluid out, eGFR.
